# *Pisolithus arhizus* Inoculation Enhances Mercury Tolerance and Growth of *Trifolium repens* in Mining Soils

**DOI:** 10.3390/toxics14080716

**Published:** 2026-08-13

**Authors:** Mónica López Velarde Santos, Adrian Ferrucio García-Morales, José Alberto Rodríguez Morales, Felix Leao Rodríguez Fierros, Yesenia Mendoza-Burguete, Juan Campos-Guillén, Miguel Angel Ramos-López, María del Carmen González-López, María Carolina Espinosa Arzate, Luisa Ramírez Granados, Ricardo Chaparro Sanchez

**Affiliations:** 1 Facultad de Ingeniería, Universidad Autónoma de Querétaro, Cerro de las Campanas S/N, Las Campanas, Querétaro 76010, Mexico; monica.lopez.velarde.santos@uaq.mx (M.L.V.S.); agarcia89@alumnos.uaq.mx (A.F.G.-M.); maria.yesenia.mendoza@uaq.mx (Y.M.-B.); maria.espinosa@uaq.mx (M.C.E.A.); luisa.ramirez@uaq.mx (L.R.G.); 2 Facultad de Ciencias Naturales, Universidad Autónoma de Querétaro, Av. de las Ciencias S/N, Delegación Juriquilla, Querétaro 76230, Mexico; 3 Facultad de Química, Universidad Autónoma de Querétaro, Cerro de las Campanas S/N, Las Campanas, Querétaro 76010, Mexico; juan.campos@uaq.mx (J.C.-G.); miguel.angel.ramos@uaq.mx (M.A.R.-L.); 4 Facultad de Química, Universidad Autónoma de Querétaro, Av. Panamericana 180 int. 100, Centro 7670 Pedro Escobedo, Querétaro 76010, Mexico; maria.delcarmen.gonzalez@uaq.mx; 5 Facultad de Informática, Universidad Autónoma de Querétaro, Av. de las Ciencias S/N, Delegación Juriquilla, Querétaro 76230, Mexico; rchapa@uaq.mx

**Keywords:** mercury, mining soils, mycoremediation, *Pisolithus arhizus*, soil toxicity, *Trifolium repens*

## Abstract

**Highlights:**

**What are the main findings?**

- Inoculation with the ectomycorrhizal fungus *Pisolithus arhizus* significantly enhanced the growth, biomass production, and mercury (Hg) tolerance of *Trifolium repens* (white clover) in contaminated mining soils.
- Mycorrhized plants achieved a 9.53% mercury removal rate from the soil, compared to a 4.33% removal rate in non-mycorrhized treatments.
- Inoculated plants successfully accumulated nearly 9% of soil Hg, yielding a Bioconcentration Factor (BCF) of 0.939, which classifies *T. repens* as an Hg low accumulator under these experimental conditions.

**What are the implications of the main findings?**

- The study demonstrates that mycorrhizal symbiosis functions as a “metabolic buffer,” offering a viable, nature-based strategy to mitigate the phytotoxic effects of heavy metals and support more sustainable land rehabilitation practices in mining-impacted areas.
- The delayed growth recovery observed in contaminated treatments highlights that the symbiotic benefit is contingent upon the establishment of an extensive extraradicular mycelial network, identifying *P. arhizus* as a critical bio-amendment for ecosystem restoration.

**Abstract:**

While mining provides critical raw materials for modern infrastructure and the energy transition, it leaves behind severe environmental damages, such as soil mercury (Hg) contamination. In the present study, we present the first study evaluating the relationship of the ectomycorrhizal fungus *Pisolithus arhizus* with *Trifolium repens* (white clover) as a novel nature-based mycoremediation strategy for mercury-stressed soils. Through a controlled 2 × 2 factorial experiment over six months, we demonstrate that *P. arhizus* significantly enhances both plant biomass and heavy-metal tolerance. Inoculation achieved a 9.53% reduction in soil Hg and drove a dramatic 53% increase in stem growth under Hg stress (compared to 36% in non-contaminated conditions) once the extraradicular mycelial network was established. Furthermore, we report novel soil-chemistry dynamics under Hg stress, including a 16% shift in electrical conductivity alongside unexpected increases in the available potassium (+9%) and phosphorus (+3%). These findings present first results for a novel approach of mycoremediation by incorporating *P. arhizus* into *T. repens* co-cultivation as a viable remediation strategy, prior to field-scale remediation.

## 1. Introduction

Soil contamination by heavy metals, particularly mercury (Hg) from industrial activity and fossil fuel combustion, is a major environmental and public health crisis. Anthropogenic inputs have raised soil Hg levels 3 to 10 times above baselines [1,2,3]. In mining hotspots like San Joaquín, Querétaro, Mexico, 46% of soil samples exceed regulatory safety limits (23 mg·kg^−1^), leading crops like maize to absorb dangerous Hg levels that threaten the food chain [3,4,5]. Conventional physical and chemical remediation such as soil excavation, washing, and thermal desorption, are often expensive, energy-intensive, and destructive to the soil structure and microbial ecology [6,7]. Biological remediation strategies present a sustainable, cost-effective alternative. Mycoremediation leverages fungal symbioses to enhance nutrient acquisition, N (nitrogen) and P (phosphorus), alleviate abiotic stress, and restore soil quality [8,9]. Ectomycorrhizal fungi (EcMs) such as *Pisolithus arhizus* are particularly promising due to their natural resilience in high-metal, low-pH, and nutrient-deprived soils [10,11,12,13]. *P. arhizus* secretes lignin- and phenol-degrading enzymes, modulates host physiology under extreme contamination, and consistently improves plant survival and nutritional status across degraded sites. Actually, *P. arhizus* is normally found in soils with high contamination levels, harsh environments, low organic matter content, and low pH [10,11,12,13,14]. Concurrently, the legume *Trifolium repens* (white clover) is widely utilized in phytoremediation owing to its rapid growth rate, high biomass production, and robust antioxidant system, which buffers reactive oxygen species (ROS) under heavy-metal stress. While *T. repens* has demonstrated high tolerance and accumulation capacity for metals such as cadmium, lead, and zinc in moderately contaminated soils, its performance and phytotoxicity thresholds under severe Hg stress remain constrained when unassisted [15,16,17].

Despite the well-documented individual phytoremediation capacities of *P. arhizus* and *T. repens* [10,14,15,16,17], significant research gaps persist in the current literature. First of all, the unexplored symbiotic pairing under mercury stress is addressed. Although previous studies on *P. arhizus* have primarily focused on organic pollutants or other heavy metals, e.g., Cd (cadmium), Pb (lead), Zn (zinc), its functional interaction with *T. repens* in mercury-polluted substrates remains unexamined. Secondly, the mechanistic insights in real mining soils are poorly understood. Mechanisms through which *P. arhizus* modulates host plant nutrient activity, and Hg compartmentalization when exposed to complex, field-sourced mining soils (rather than spiked synthetic media) should be thoroughly understood. Finally, the limitations of single-organism approaches should be overcome. Monospecific phytoremediation using *T. repens* alone often suffers from growth inhibition under high Hg exposure, while mycoremediation without a fast-growing host plant lacks sufficient biomass production for ecosystem stabilization.

To address these literature gaps, this study evaluates the simultaneous performance of *P. arhizus* and *T. repens* in mercury-impacted mining soils sourced from San Joaquín, Querétaro. The primary objective of this work is to determine the efficiency of *P. arhizus* inoculation in enhancing the growth, nutrient uptake (N and P), and Hg tolerance of *T. repens*. The scope of the study encompasses controlled soil–plant experiments measuring plant growth, nutritional status, and mercury translocation dynamics within soil concentrations exceeding environmental threshold limits. Pilot-scale field validations must be carried out to prove efficacy over a long period in real-world conditions.

The significance of this study lies in pairing a resilient EcM fungus with a high-biomass leguminous host. This research introduces a novel, dual-action mycoremediation framework. The findings provide insights into fungus–plant–metal interactions, offering first results of a possible, scalable, and ecological biotechnology for restoring heavily degraded mining sites in Latin America and similar mining ecosystems worldwide.

## 2. Materials and Methods

### 2.1. Study Area and Soil Collection

**Figure 1:**
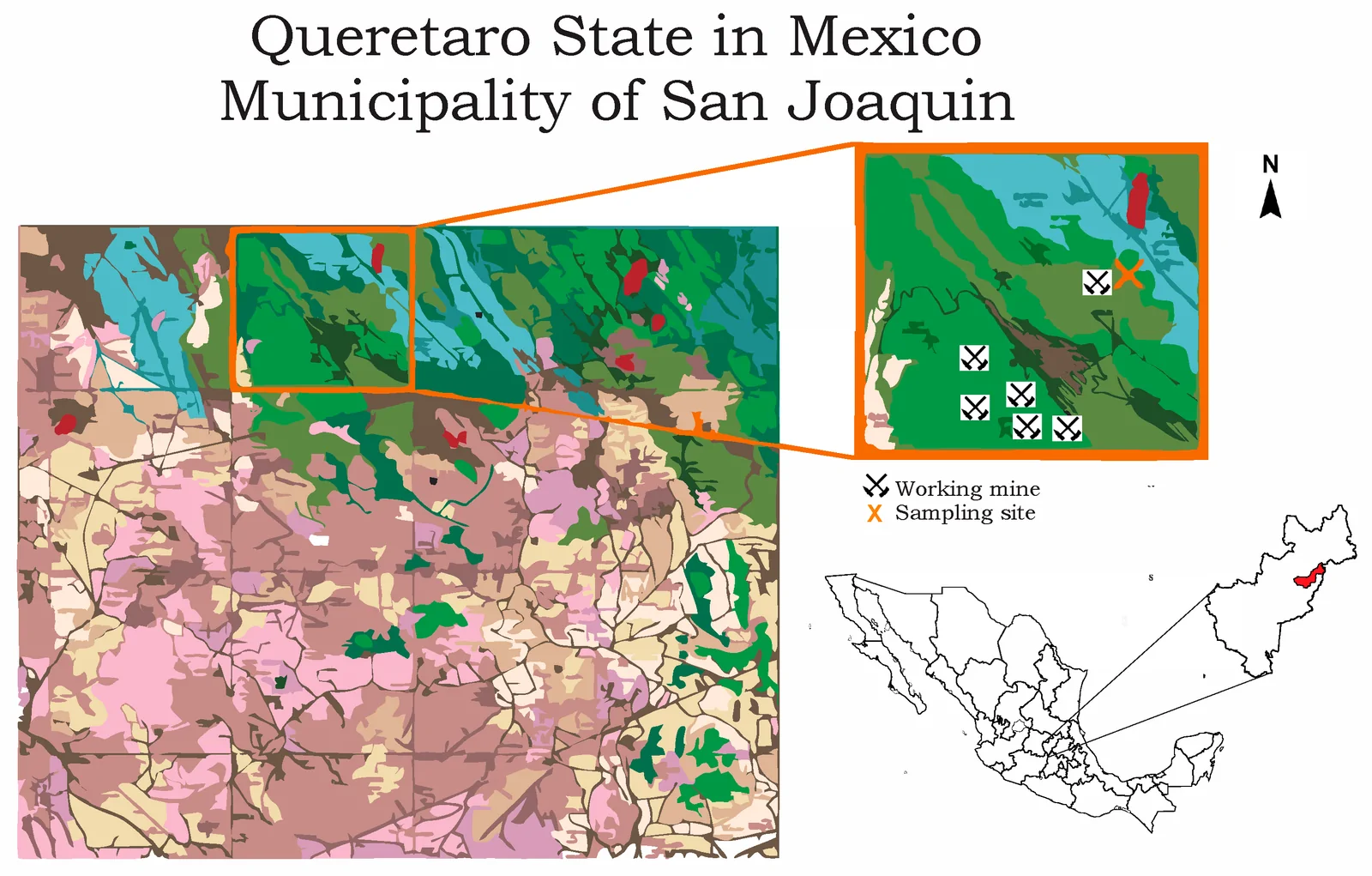
Location of the soil collection area in the municipality of San Joaquín, Querétaro, Mexico (X marks the sampling site), https://mapserver.sgm.gob.mx/Cartas_Online/geologia/88_F14-11_GM.pdf (accessed on 5 August 2026).

Soil samples were collected from the San Joaquín municipality in the Southern Sierra Gorda Queretaro, Central Mexico located at a longitude of 99°37′ W and a latitude of 20°55′ N (Figure 1). Sampling followed the NMX-AA-132-SCFI-2016 standard [18], which dictates the procedures for the identification and quantification of metals and metalloids in soil. Standard NMX-AA-132-SCFI-2016 [18] specifies sampling at the 0–5 cm depth range for the surface layer because it aims to assess the risk of acute, direct exposure for humans. Contaminants could be concentrated at this shallow depth, which is also where the greatest potential for dermal contact and dust inhalation exists. Exploratory sampling was performed at this depth, taking 6 field subsamples from vertical soil profiles using a clean stainless-steel spade. Samples were transported to the laboratory in sealed plastic bags. In the laboratory, soil samples were mixed and homogenized by grinding and sieving through a 3 mm mesh. Samples were stored at 4 °C in clean plastic bags for less than 24 h before the assays were prepared.

### 2.2. Soil Characterization

The initial characterization of the uncontaminated nursery soil and the contaminated mining soil mix is shown in Table 1. Hg concentrations in the soil and in *T. repens* tissues were quantified using the methodology described below. Additionally, weekly recordings were made for N, P, and potassium (K) levels (mg·kg^−1^), electrical conductivity (EC, µS·cm^−1^), pH, temperature, and humidity, with the high-precision environmental sensor JXBS-3001-SCY-PT, from Mayubao.

### 2.3. Experimental Design

A 2 × 2 factorial design was implemented, evaluating two primary factors, soil (X) and mycorrhizal inoculation (Y). Each factor was tested at two levels: without (sub-index 0) and with (sub-index 1) the respective treatment. The experimental matrix is detailed in Table 2. To ensure the statistical significance of the results, three biological replicates for each experimental group or treatment were prepared.

A total of 12 rigid polypropylene (PP) pots were prepared, each containing 500 g of soil, with dimensions of 12 cm diameter and 12 cm height. Six pots were filled with 450 g of homogenized nursery soil and defined as control treatments (X_0_Y_0_ and X_0_Y_1_). To account for any physiological or nutritional effects caused by the inoculum-carrier matrix itself, an inoculum-carrier control group was established. This control received an equivalent amount of sterilized, uncolonized carrier substrate (free of *Pisolithus arhizus* mycelium) under identical preparation and handling conditions, ensuring that any observed plant response can be strictly attributed to the fungal symbiosis rather than the carrier components.

The other 6 pots were filled with field-collected soil mixed at a 1:1 ratio (*v*:*v*) with clean nursery soil. The contaminated treatments were named X_1_Y_0_ and X_1_Y_1_. Only one mercury concentration was tested, in case the achieved results met acceptable remediation rates. Further analyses with increasingly contaminated soil content will be a subsequent consideration.

The experiment was conducted under controlled shade-house conditions from June to November 2025. Pots were arranged in a completely randomized design on benches, maintaining a uniform spacing of 20 cm between containers to avoid microclimatic shading effects. Natural sunlight was supplemented to maintain an average photosynthetically active radiation (PAR) light intensity of 450 ± 25 µmol m^−2^ s^−1^ under a fixed 12 h photoperiod. Soil moisture was monitored daily using a capacitive moisture sensor, maintaining a target volumetric water content (VWC) between 60% and 70% of field capacity. Irrigation was supplied uniformly every 48 h using a standardized volumetric watering protocol (150 mL per pot per watering event) with distilled water to prevent additional ionic interference, while depth rationale and temperature/relative humidity (RH) logging were kept constant as previously described.

### 2.4. Trifolium repens

*Trifolium repens* belongs to the plant kingdom and comprehends about 300 species of flowering plants from the Fabaceae family. It is one of the most important legumes cultivated as forage crops. It fixes nitrogen in the agricultural fields and reaches a height of around 10 cm, with a 3-leaflet pattern and white flower heads.

To prepare the assay, small gray seeds with a 1 mm diameter of *T. repens* were obtained vacuum-packed and were propagated in the pots, according to the experimental design. Seeds of *Trifolium repens* (white clover, cv. Huia, lot number TR-2025-04, with a certified germination percentage of 92%) were purchased from Agro-Semillas S.A. de C.V. (Querétaro, Mexico). Prior to sowing, seeds were surface-sterilized by immersion in 70% ethanol for 2 min, followed by 1% sodium hypochlorite for 5 min, and thoroughly rinsed five times with sterile distilled water, according to Báez-Pérez et al. [8]. In every pot, 5 g of seeds were homogeneously spread, at a depth of 1 cm. A soil layer (1 cm) was carefully removed with a spatula spoon, seeds were homogenously placed, and soil was again placed thereon.

### 2.5. Mycorrhiza

MycoRacine MP from MYCOBIOSFERA containing *Pisolithus arhizus*, Vermiculite, and Diatomaceous earth was used as inoculant. It was delivered in a rough powder form of brown color. According to the specifications of the supplier, it consisted of 525 × 105 spg (spores per gram). *Pisolithus arhizus* is a widespread, ectomycorrhizal gasteroid fungus belonging to the fungi kingdom, from the basidiomycota division, agaricomycetes class, and sclerodermataceae family. When grown, *P. arhizus* has a bizarre appearance and historical use in natural dyeing. It forms vital symbiotic relationships with various trees.

The experimental pots X_0_Y_1_ and X_1_Y_1_ were filled roughly two-thirds with soil and tamped down gently to create a stable surface. A uniform, concentrated layer of 3 g *P. arhizus* spores was placed across the substrate surface. Afterwards, the inoculum was covered through a soil layer of 1 cm, and half a fistful of *Trifolium repens* seeds was placed above the last layer.

Spore viability was tested with a hemocytometer, after the spore suspension was dyed with trypan blue 0.05%. Dead spores absorb the dye and become blue, while live spores exclude the dye and become clear. To count the spores, the hemocytometer with the dyed spore solution was observed with an optical microscope (100×–400×) [19,20]. The total live spores were divided by the sum of total dead and live spores.

After 22 weeks of experiments, mycorrhization was evaluated by dyeing mycorrhized roots with trypan blue according to Báez–Pérez et al. [21] and Thomson et al. [22], in order to compare the mycorrhization of each treatment, examining one root fragment for each pot. Trypan blue solution was prepared at 0.05%. Roots were cleaned with distilled water, dyed abundantly with trypan blue, and left for 48 h at 45 °C temperature. Afterwards, the trypan blue solution was washed with lactoglycerol, and the root fragments were observed with an optical microscope (100×–400×).

### 2.6. Hg Determination

Hg concentrations were determined in whole plant tissues via acid digestion following the NMX-AA-051-SCFI-2016 standard [23]. Acid digestion was performed using the EPA Method 3050B [24] (nitric acid, hydrochloric acid, and hydrogen peroxide) to convert Hg into its ionic form. At this point, samples were sent to an external laboratory called “Centro de servicios quimicos”, where Hg concentration was measured with an atomic absorption spectrophotometer (AAS) and expressed on a dry-weight basis. The calibration procedure for the Hg measurement included serially diluting a traceable Hg stock solution to bracket the expected sample concentration range including 5 calibration points (0.5 to 10 μg/L). A matrix-matched blank with deionized water was prepared. A hydride generator was used. The liquid sample was treated with a reducing agent to convert the mercury into a gas. This vapor was carried by a flow of inert gas (argon) into a quartz cell located in the AAS equipment. The measurement was performed flameless at a specific wavelength of 253.7 nm.

According to the NMX-AA-051-SCFI-2016 standard [23], each laboratory that uses this method must operate a formal quality control program described in the norm.

### 2.7. Bioconcentration Factor

The metal bioconcentration factor (BCF) was calculated according to the following equation, based on dry weight [15].

$$BCF=\frac{C\;plant}{C\;soil}$$

C plant stands for the concentration in mg·kg^−1^ of the HM in the plant, and C soil for the same in the soil. According to Sulaiman and Hamzah [25], values of BCF > 1 indicate that the plant is a hyperaccumulator, ≤1 a moderate accumulator, ≤0.1 a low accumulator, and 0.01 a non-accumulator.

### 2.8. Stem and Leaf Measurements

Stem and leaf growth of *T. repens* was measured weekly. Measurements were carried out on Monday morning and were recorded in a laboratory logbook. Measurements were taken with a Vernier caliper. In each pot, the ten plants with the highest growth were chosen, and the average value was recorded. Stem length was measured from the soil surface to the apical tip/highest node, and leaf diameter was recorded as the maximum width of the leaf blade.

### 2.9. Statistical Analysis

Regarding leaf and stem growth, data were analyzed using repeated-measures ANOVA to evaluate the interaction between time, Hg exposure, and mycorrhizal inoculation. Repeated-measures ANOVA is performed to compare the means of groups measured over time. To corroborate the assumptions resulting from the ANOVA, a post hoc Tukey pairwise comparison was performed with a confidence interval of 95%.

To compare the interactions between Hg and mycorrhiza in the parameters measured (N, P, K, EC, pH, temperature, and humidity), a two-way ANOVA followed by a Tukey pairwise comparison was performed.

To obtain a statistical comparison of Hg content, 2-sample *t* tests were performed between treatments (with and without mycorrhiza). At first, a normality test according to Ryan–Joiner (similar to Shapiro–Wilk) was performed, and the homogeneity of variances was tested.

All tests (*T. repens* growth, Hg determination, and parameters measured) were performed in triplicate as three different individual plants or measurements (*n* = 3). Statistical analyses were performed using Minitab 17.1.0.

## 3. Results

**Figure 2:**
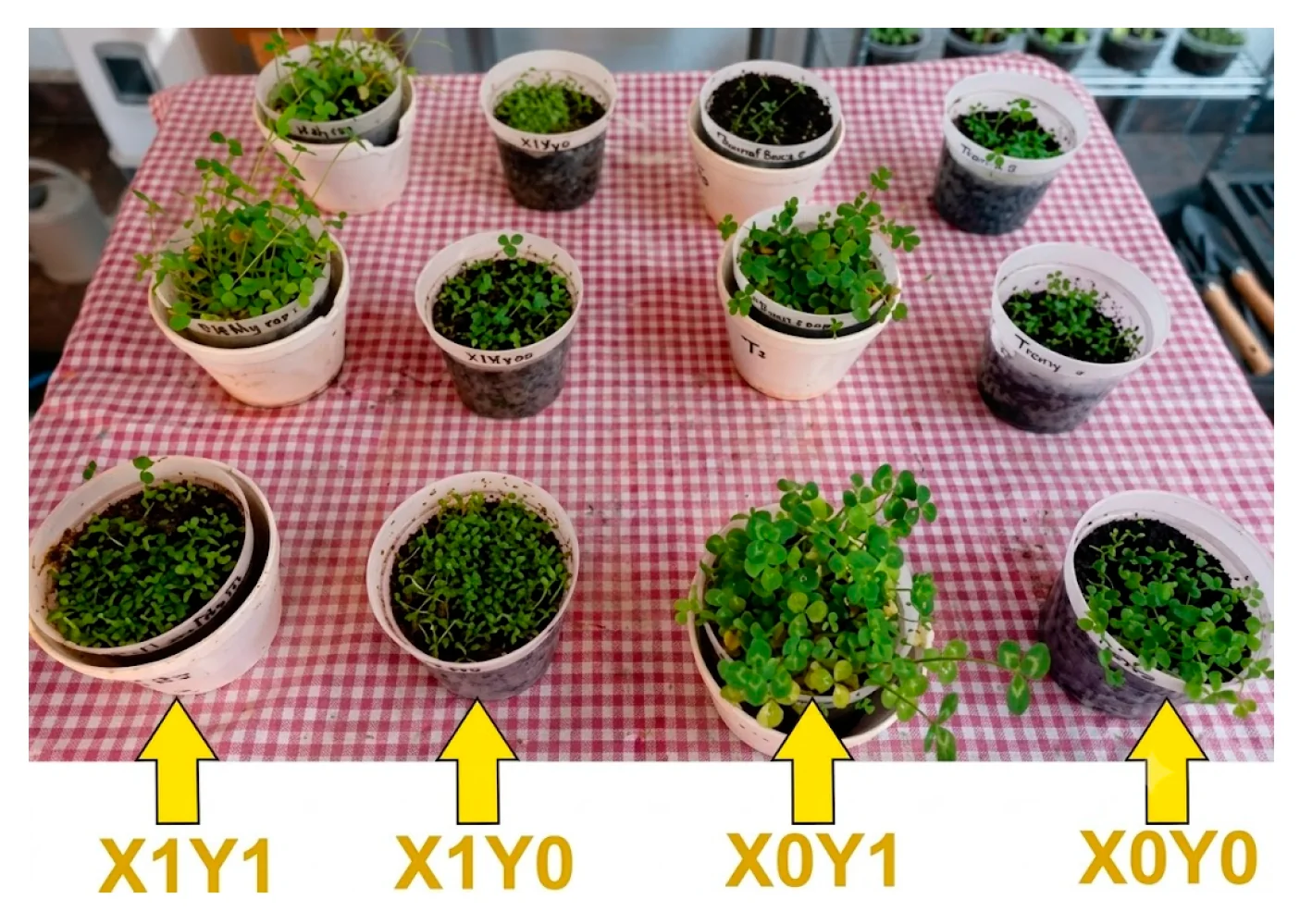
Photograph of the 22-week experimental trial with triplicate pots for each treatment combination. Legend: X_0_ = uncontaminated nursery soil; X_1_ = contaminated mining soil; Y_0_ = non-inoculated; Y_1_ = mycorrhizal inoculated.

The inoculation of *T. repens* with the ectomycorrhizal (EcM) fungus *Pisolithus arhizus* was successful across all inoculated treatments. Visual inspection at the conclusion of the 22-week experiment revealed a distinct difference in *T. repens* growth between mycorrhized (Y_1_) and non-mycorrhized (Y_0_) treatments (Figure 2).

### 3.1. Hg Content

The mercury concentration levels recorded across the triplicates of the four treatments are summarized in Table 3. The initial Hg concentration in the contaminated treatments (X_1_Y_0_ and X_1_Y_1_) was 3290 ± 13.2 mg·kg^−1^. After 22 weeks, soil Hg removal was 4.33 ± 0.2% in non-mycorrhized soil (X_1_Y_0_) and 9.53 ± 0.3% in mycorrhized soil (X_1_Y_1_). The statistical analysis showed a normal data distribution according to the Ryan–Joiner test and a homogeneity of variance; thus, a two-sample *t* test could be performed for both Hg removal and Hg soil concentration. The results of the *t* test showed that the percentage of Hg removal from the soil has a highly significant difference between treatments. For Hg removal, T-Value = 24.36; *p*-Value = 0.000; and DF = 3; and for Hg soil concentration, T-Value = −14.71; *p*-Value = 0.001; and DF = 3. Mycorrhizal inoculation significantly increased metal removal, compared to the control treatment without mycorrhiza.

Regarding plant uptake, *T. repens* inoculated with *P. arhizus* (X_1_Y_1_) absorbed 280 mg·kg^−1^ of Hg, compared to 120 mg·kg^−1^ in non-inoculated plants (X_1_Y_0_). Consequently, the bioconcentration factor (BCF) for the inoculated treatment was 0.093, whereas the non-inoculated treatment yielded a BCF of 0.038. Following the criteria proposed by Sulaiman and Hamzah [25], these values classify the mycorrhizal symbiosis between *T. repens* and *P. arhizus* as a low Hg accumulator in this experimental context.

### 3.2. T. repens Growth

**Figure 3:**
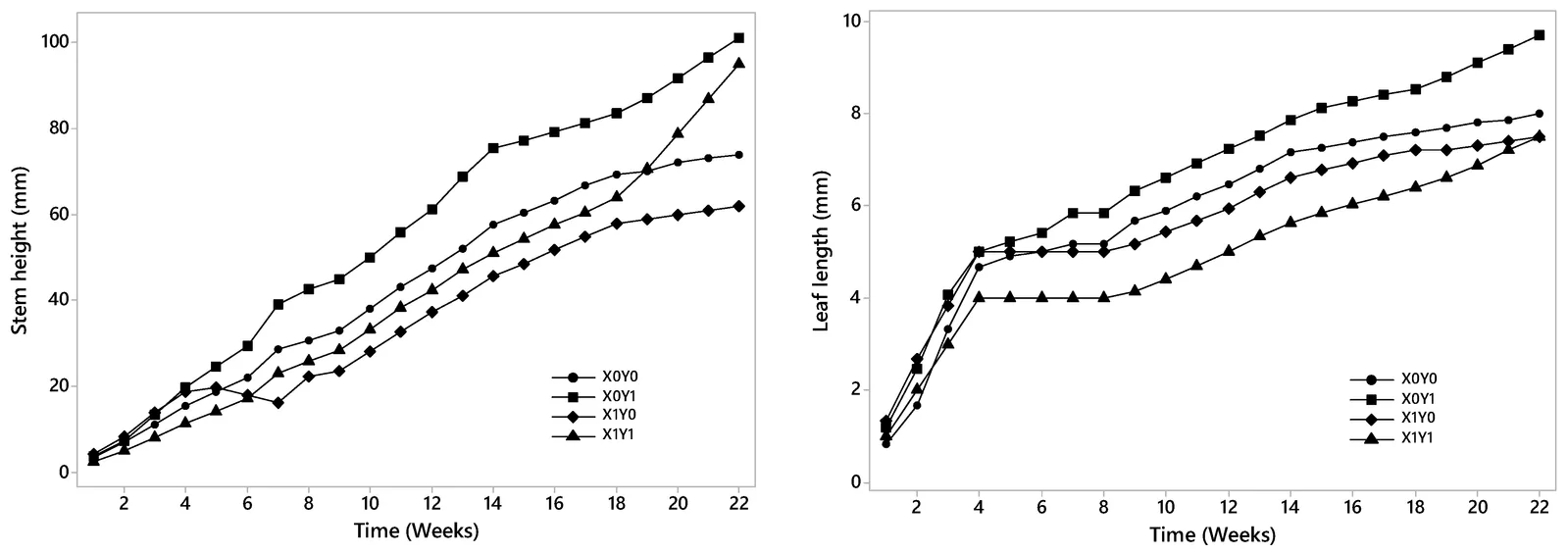
Stem height (**left panel**) and leaf length (**right panel**) of *T. repens* after 22 weeks from triplicate treatments.

Figure 3 shows the average growth of *T. repens* regarding stem height (left panel) and leaf length (right panel) from the triplicates of the four treatments. As shown in the left panel of Figure 3, clover grown in uncontaminated soil inoculated with mycorrhiza (X_0_Y_1_) showed the highest stem growth. *T. repens* grown in contaminated soil inoculated with mycorrhiza (X_1_Y_1_) showed a low growth until week 18, afterwards surpassing the growth curves of *T. repens* without mycorrhiza (X_0_Y_0_ and X_1_Y_0_), and approximating *T. repens* grown in uncontaminated soil with mycorrhiza (X_0_Y_1_). The lowest stem growth was exhibited by the plants grown in contaminated soil without mycorrhiza (X_1_Y_0_).

Similarly to stem growth, leaf growth was higher for the treatment with X_0_Y_1_, non-contaminated mycorrhized soil (right panel of Figure 3). As expected, leaf growth was lower for the treatments with contaminated soil (X_1_). Nevertheless, after 22 weeks of experiments, the treatments with contaminated soil (X_1_Y_1_ and X_1_Y_0_) reached similar values.

The *p*-Values obtained from the repeated-measures ANOVA in stem and leaf growth were *p* = 0.000 for both factors, treatment and time.

Table 4 shows the pairwise comparison with the Tukey method for stem and leaf growth. Treatment and time were compared, and growth was the response. A significant difference between almost all means can be appreciated, except between X_1_Y_0_ and X_0_Y_0_ in the case of leaf growth (*p*-Value = 0.208), and X_1_Y_1_ and X_0_Y_0_ in the case of stem growth (*p*-Value = 0.702). After week 18, stem height in contaminated mycorrhized clover achieved similar values compared with treatments without inoculation in non-contaminated soil.

The weekly monitoring of stem and leaf growth provided insight into the stress-mitigation capacity of *P. arhizus*. Regarding stem growth, as shown in Figure 3 and the Tukey analysis (Table 4), the X_0_Y_1_ treatment (uncontaminated, mycorrhized) exhibited the highest growth rates. Interestingly, plants in the X_1_Y_1_ group showed slow growth until week 18, after which they accelerated, surpassing the growth curves of both X_1_Y_0_ and X_0_Y_0_ treatments. Regarding leaf growth, leaf length was significantly higher in X_0_Y_1_. The statistical analysis confirmed that the X_1_Y_1_ treatment was significantly different from X_0_Y_1_. While contamination (X_1_) generally depressed leaf growth, the mycorrhizal symbiosis in X_1_Y_1_ allowed for recovery, leading to final values comparable to non-contaminated controls.

### 3.3. Physicochemical Soil Parameters

The contents of N, P, and K (mg·kg^−1^), as well as the EC (µS·cm^−1^), humidity (%), temperature (°C), and pH, were measured daily in all triplicates of the four treatments. A Tukey pairwise comparison with a 95% confidence interval was performed to find the effects that the presence of Hg and mycorrhiza had on the measured parameters. Table 5 shows the results of the Tukey test. The adjusted *p*-Values showing <0.05 do reflect a statistically significant difference for the treatments containing mercury. K, P, and EC show a difference among treatments with (X_1_) and without (X_0_) contaminated soil.

The results suggest that Hg contamination, rather than the mycorrhizal inoculation itself, could be the primary factor driving the shifts in K and P availability, as well as soil conductivity. These results underscore the severity of the mining-derived Hg contamination in altering the chemical microenvironment of the soil.

## 4. Discussion

### 4.1. T. repens Growth Response

The results indicate that the highest vegetative development, in terms of both leaf and stem growth, occurred in the non-contaminated, mycorrhized treatment (X_0_Y_1_). Conversely, plants grown in contaminated soil without inoculation (X_1_Y_0_) exhibited the most restricted development. This growth enhancement mediated by mycorrhizal fungi is consistent with findings reported by multiple researchers [26,27,28,29,30]. Fiqri et al. [28] observed similar patterns in maize, where mycorrhizal inoculation facilitated growth in soils post-mercury phytoextraction.

Comparative analysis of plant growth metrics reveals distinct patterns between foliar expansion and stem elongation. While leaf growth was predominantly dictated by the presence of mercury (Hg) in the substrate, stem development showed a more pronounced sensitivity to the combined effects of Hg contamination and mycorrhizal inoculation. These findings align with the physiological roles of these tissues: leaves serve as primary sites for photosynthetic capture, whereas stems are responsible for long-distance nutrient and water transport. Previous research indicates that mycorrhizal symbiosis enhances photosynthesis by optimizing plant nutrient status [31,32,33]. In our experiment, the proximity of the stem to the root system—the primary interface for *P. arhizus* colonization—suggests that the mycorrhizal influence on nutrient translocation is more directly manifested in stem elongation than in leaf surface area. This preferential allocation of resources towards root and stem development is consistent with established models where mycorrhizae reconfigure plant resource distribution under metal-induced stress [31,33,34]. The delayed growth recovery observed in contaminated, inoculated treatments (X_1_Y_1_) suggests that the symbiotic benefit is contingent upon the establishment of the extraradicular mycelial network. Our results differ from studies reporting that mycorrhizae exclusively mitigate heavy-metal (HM) stress by immobilizing Hg within root tissues [35,36]. This discrepancy may arise from our experimental design, where inoculation occurred simultaneously with seed germination, as opposed to the use of pre-germinated seedlings or exogenous amendments like humic acids commonly found in the literature. Furthermore, we observed limitations in leaf growth despite mycorrhiza presence, which may be explained by the mechanism proposed by Hachani et al. [29]. The presence of high concentrations of metallic ions can saturate specific ion transport channels, thereby inhibiting the uptake of essential nutrients required for efficient foliar development. Our data confirm that Hg contamination exerts a significant detrimental effect on stem growth, perhaps due to structural damage to the vascular system and transport tissues [37,38,39,40]. This topic should be researched further to verify this statement. While soil contamination poses an undeniable stress factor, the presence of *P. arhizus* could facilitate the necessary adaptability for *T. repens* to resist Hg-induced impairment once the symbiotic equilibrium is established. Escobar-Vargas et al. [41] demonstrated that mycorrhizal inoculation not only promotes seedling growth in metal-contaminated substrates but also protects germination indices from the toxic effects of mercury.

### 4.2. Soil Nutrient and EC Changes

Our results indicate that mercury (Hg) presence, rather than mycorrhizal inoculation, is the primary driver of changes in soil EC and the concentration of K and P. Specifically, contaminated soils showed significant increases of 27% in EC, 20% in K, and 14% in P. As reported by several authors, this elevation in EC could be attributed to the displacement of soil cations, e.g., Ca^2+^, Mg^2+^, or K^+^, by ionic mercury (Hg^2+^) during cation exchange, which increases the concentration of dissolved ions in the soil solution [31,42]. Furthermore, authors have reported that the production of organic acids by mycorrhizae may inadvertently facilitate the dissolution of soil minerals into ionic forms, further contributing to higher EC values [43,44]. This statement could be further researched to clarify the topic.

Regarding N content, no significant differences were detected between treatments. This contrasts with findings by Szada-Borzyszkowska et al. [45], who reported a 13.5% reduction in N content in mycorrhized Miscanthus under Hg stress. Similarly to the N behavior, no significant variations in pH or substrate humidity were observed, consistent with reports by Escobar-Vargas et al. [41] regarding inoculated lettuce.

The observed increase in soil K and P can be explained with findings from other authors, suggesting that Hg-induced oxidative damage to root cell membranes may inhibit the plant’s metabolic capacity for nutrient uptake. It is hypothesized that the binding of Hg to sulfur-containing proteins and enzymes disrupts metabolic pathways essential for P and N absorption, leaving these nutrients in the soil rather than within the plant tissues [45,46,47,48]. This differs from the results of Liu et al. [48] and Hachani et al. [29], who observed that mycorrhization significantly improved P uptake in Aleppo pines via the secretion of organic acids like oxalate. Finally, the shift in K and P availability may also be linked to alterations in the soil microbial community. According to Couic et al. [49], the presence of trace elements in soils, such as As (arsenic), Cd, Cu (copper), Pb, or Hg, can alter microbial communities and their growth, affecting the mineralization of C (carbon) and N. Additionally, our successful mycorrhizal colonization, evidenced by hyphal penetration, suggests that *T. repens* maintains symbiotic integrity even in the presence of Hg. To have a better understanding, a deeper analysis should be carried out regarding the metabolic pathways for nutrient absorption.

Statistical analyses of soil physicochemical parameters revealed that mercury contamination acts as the primary driver governing shifts in electrical conductivity (EC) and available potassium (K) and phosphorus (P), rather than the mycorrhizal inoculation itself. The ionic displacement caused by mercury in the soil matrix triggers these chemical variations, whereas the symbiont’s main contribution focuses on protecting plant physiological pathways against this harsh chemical disruption rather than directly dictating baseline soil ion fluctuations.

### 4.3. Hg Uptake/Removal

After 22 weeks of experimentation, a reduction in soil mercury (Hg) content was observed, with mycorrhized *T. repens* exhibiting a 9.42% removal rate compared to 4.25% in non-mycorrhized treatments. Hg quantification was performed only in the soil and in the whole plant tissue. Several authors consider *T. repens* as a good metal accumulator; nevertheless, in this assay, BCF calculations showed contrary results with a BCF ≤ 0.1.

Mycorrhizal inoculation increased metal removal, compared to the control treatment without mycorrhiza. This reduction, coupled with the observed increase in electrical conductivity, supports the findings of Debeljak et al. [27], who suggest that mycorrhizae enhance Hg mobility and bioavailability in the soil matrix. Furthermore, some authors report that mycorrhizal inoculation confers HM tolerance by increasing the root surface area, facilitating the immobilization of metals within root tissues [50,51,52].

Regarding the role of the symbiont, Escobar-Vargas et al. [41] posit that mycorrhizae filter out toxic HMs by sequestering them within fungal cell walls, cytoplasmic granules, or glomalin, thereby preventing translocation into plant tissues. This suggests that the primary contribution of mycorrhizae to plant integrity may lie in improving nutritional conditions rather than solely preventing cellular damage caused by Hg. Furthermore, Fiqri et al. [28] observed that arbuscular mycorrhizal (AM) fungus inoculation did not significantly alter Hg accumulation in species such as *P. conjugatum*, *C. kyllingia*, and *L. crustacea*, suggesting that the observed loss of Hg in soil may, in some cases, be attributed to volatilization facilitated by the presence of AM fungi.

Although *T. repens* is widely recognized in the literature as a potential metal-accumulating species, the bioconcentration factor (BCF) values obtained in this 22-week assay (≤0.1) classify the mycorrhizal system strictly as a low accumulator under these specific experimental conditions. Therefore, rather than acting as a high-efficiency phytoextractor, the primary role of *P. arhizus* lies in conferring heavy-metal tolerance and facilitating a controlled immobilization apparently within the root–mycelium complex while promoting localized soil mercury reduction.

In further analyses regarding mycoremediation for Hg removal, mass balance should be calculated to track the real metal transformation from a dissolved liquid state into an insoluble and stable solid state.

### 4.4. Study Limitations

The present study has several limitations that should not be overlooked. While laboratory experiments in controlled environments provide excellent systematic insights, translating those results to the real world is notoriously difficult. Real-world contaminated soils contain highly complex forms of mercury at different concentrations or root/shoot ratios. Moreover, to isolate the specific fungal mechanism, sterile soil is used in laboratory assays to avoid outside interference. At the same time, the native microbiome eliminates real-world variables like competitive exclusion or synergistic co-metabolism. In addition, experimental setups utilizing small pots or Petri dishes apply artificial boundaries on root and hyphal development, altering the real contact time between the mycelium and Hg contamination, and leading to unreal accumulation rates that do not scale to open-field environments. The next step should be to conduct an in situ analysis of Hg mycoremediation to simulate mercury removal in practice. Through phytoextraction, the total volume of contaminants (HMs) can be reduced in the soil, where plants absorb HM through the roots, transporting them to the stems and leaves. The above-ground plant parts can be frequently harvested and incinerated as hazardous waste, slowly cleaning the site over repeated cycles.

A feasibility study together with a life cycle assessment could be carried out, to determine the economical, technical, and operational viability of the remediation strategy, considering its environmental impacts.

## 5. Conclusions

In conclusion, while *Pisolithus arhizus* inoculation significantly enhances *Trifolium repens* growth under mercury stress—particularly promoting stem elongation after week 18—the system operates primarily as a low accumulator (BCF ≤ 0.1) rather than a hyperaccumulator. Soil property alterations (EC, K, and P) were predominantly driven by mercury contamination dynamics rather than the fungal presence. These findings demonstrate that the success of the symbiosis relies on imparting stress tolerance and enabling moderate soil remediation (9.53% removal), highlighting the need for pilot-scale field validations to assess long-term operational feasibility.

## Figures and Tables

**Table 1:** Baseline physicochemical characterization of the uncontaminated nursery soil and the contaminated mining soil mix used in the experiment. Data are shown as mean ± standard deviation.

Parameter	Uncontaminated Nursery Soil	Contaminated Mining Soil Mix
Texture	loam	silt
pH	6.38	6.81
Electrical conductivity (µS·cm^−1^)	95.33 ± 8.5	253.33 ± 14.15
Humidity (%)	2.3 ± 0.2	8.5 ± 0.6
Nitrogen (mg·kg^−1^)	6.33 ± 0.58	18 ± 1.63
Phosphorus (mg·kg^−1^)	9 ± 0.82	25.33 ± 2.10
Potassium (mg·kg^−1^)	18.66 ± 1.45	51 ± 3.27
Mercury (mg·kg^−1^)	0	3290 ± 13.2

**Table 2:** Factorial experimental design (2 × 2) showing treatment combinations of soil type and mycorrhizal inoculation. All treatments were performed in triplicate (*n* = 3).

Treatments	Factor		Replicates (*n*)
	Soil (X)	Mycorrhiza (Y)	
1	X_0_	Y_0_	3
2	X_1_	Y_0_	3
3	X_0_	Y_1_	3
4	X_1_	Y_1_	3

X_0_: uncontaminated nursery soil; X_1_: contaminated mining soil mix; Y_0_: non-inoculated; Y_1_: *Pisolithus arhizus*-inoculated.

**Table 3:** Mercury concentrations (mg·kg^−1^) in soil and *T. repens* tissues after 22 weeks. Values are presented as mean ± standard deviation.

Treatment	Initial Soil Hg (t_0_)	Residual Soil Hg (t_22_)	Hg Accumulated in *T. repens* (t_22_)
X_0_Y_0_	n.d.	n.d.	n.d
X_0_Y_1_	n.d.	n.d.	n.d.
X_1_Y_0_	3290 ± 13.2	3150 ± 12.3	120 ± 5.4
X_1_Y_1_	3290 ± 13.2	2980 ± 15.8	280 ± 8.2

X_0_: uncontaminated nursery soil; X_1_: contaminated mining soil mix; Y_0_: non-inoculated; Y_1_: *Pisolithus arhizus*-inoculated. n.d. = not detected.

**Table 4:** Comparison of Tukey simultaneous 95% CI for stem (left panel) and leaf (right panel) growth. Data are shown as mean ± standard error.

	Stem		Leaf	
	Difference in Means	Adjusted *p*-Value	Difference in Means	Adjusted *p*-Value
X_0_Y_1_ − X_0_Y_0_	12.49 ± 1.78	0.000	0.809 ± 0.108	0.000
X_1_Y_0_ − X_0_Y_0_	−7.83 ± 1.78	0.000	−0.214 ± 0.108	0.208
X_1_Y_1_ − X_0_Y_0_	−1.93 ± 1.78	0.702	−1.010 ± 0.108	0.000
X_1_Y_0_ − X_0_Y_1_	−20.33 ± 1.78	0.000	−1.023 ± 0.108	0.000
X_1_Y_1_ − X_0_Y_1_	−14.42 ± 1.78	0.000	−1.819 ± 0.108	0.000
X_1_Y_1_ − X_1_Y_0_	5.9 ± 1.78	0.000	−0.796 ± 0.108	0.000

**Table 5:** Tukey simultaneous tests for differences in means after ANOVA.

	Adjusted *p*-Value					
	Nitrogen N	Potassium K	Phosphorus P	Conductivity EC	Humidity	pH
X_0_Y_1_ − X_0_Y_0_	0.980	0.630	0.974	0.904	0.942	0.083
X_1_Y_0_ − X_0_Y_0_	0.973	0.000	0.011	0.000	0.991	0.856
X_1_Y_1_ − X_0_Y_0_	0.192	0.000	0.000	0.000	0.990	0.988
X_1_Y_0_ − X_0_Y_1_	1.000	0.000	0.038	0.000	0.821	0.393
X_1_Y_1_ − X_0_Y_1_	0.379	0.000	0.000	0.000	0.994	0.172
X_1_Y_1_ − X_1_Y_0_	0.403	0.621	0.589	0.428	0.930	0.966

The results of the Tukey tests suggest a significant difference between treatments with (X_1_) and without (X_0_) Hg, for the three affected parameters K, P, and EC.

## Data Availability

The original contributions presented in this study are included in the article. Further inquiries can be directed to the corresponding authors.

## Abbreviations

The following abbreviations are used in this manuscript:

AM	Arbuscular mycorrhiza
As	Arsenic
AAS	Atomic absorption spectrophotometer
BCF	Bioconcentration factor
C	Carbon
Ca^2+^	Ion calcium
Cu	Copper
Cd	Cadmium
CI	Confidence interval
DF	Degrees of freedom
EC	Electrical conductivity
EcM	Ectomycorrhiza
Hg	Mercury
Hg^2+^	Ion mercury
HM	Heavy metal
K	Potassium
K^+^	Ion potassium
Mg^2+^	Ion magnesium
N	Nitrogen
P	Phosphorus
PAR	Photosynthetically active radiation
Pb	Lead
PP	Polypropylene
Spg	Spores per gram
VWC	Volumetric water content
Zn	Zinc
